# Aspergillus endocarditis of the mitral valve with ventricular myocardial invasion, cerebral vasculitis, and intracranial mycotic aneurysm formation in a patient with hemophagocytic lymphohistiocytosis

**DOI:** 10.1016/j.mmcr.2018.05.001

**Published:** 2018-05-03

**Authors:** Bibin Varghese, Kevin Ting, Juan Lopez-Mattei, Cezar Iliescu, Joseph Kim, Peter Kim

**Affiliations:** a Baylor College of Medicine, 1 Baylor Plaza, Houston, TX 77030, USA; b MD Anderson Cancer Center, 1400 Pressler Street, Box 1451, Houston, TX 77030, USA; c University of Texas at Austin, 2401 Whitis Ave, Austin, TX 78705, USA

**Keywords:** Aspergillus, Endocarditis, Hemophagocytic lymphohistiocytosis, Myocardial invasion, CNS vasculitis, Mycotic aneurysm

## Abstract

Aspergillus endocarditis is a rare infection and reported mainly in immunocompromised hosts. We report a case of mitral valve aspergillus endocarditis with ventricular myocardial invasion, cerebral vasculitis and intracranial fungal aneurysm formation in a patient with hemophagocytic lymphohistiocytosis (HLH). This case illustrates the importance of prompt investigation and treatment of masses seen on an echocardiogram for rare infections such as Aspergillus endocarditis in immunocompromised patients.

## 1 Introduction

Fungal endocarditis (FE) accounts for only 1–2% of all cases of infective endocarditis (IE) [1]. Immunocompromised patients are at especially high risk for systemic fungal infections and endocarditis [2]. Aspergillus endocarditis is extremely rare and accounts for 20–25% of all fungal endocarditis; for patients with invasive disease and subsequent hematogenous spread, mortality rates may reach up to 80–90% even with treatment [3]. We report an extremely rare case of aspergillus endocarditis with left ventricular myocardial invasion, cerebral vasculitis and intracranial fungal aneurysm formation in a patient with HLH.

## 2 Case

A 65-year-old male with a history of hemophagocytic lymphohistiocytosis (HLH) status post 10 cycles of etoposide and a steroid taper of dexamethasone, pulmonary aspergillosis diagnosed by biopsy of a right lower lobe lung nodule with completion of treatment with isavuconazole for 3 months 20 days prior to day 0, and recent disseminated cytomegalovirus (CMV) on maintenance valganciclovir, was admitted with 4 days of headache, chills, and worsening delirium. On the day of admission (day 0), his temperature was 37.2 °C, blood pressure was 95/63 mmHg, pulse was 104 beats per minute, respiratory rate was 18 breaths/min, and oxygen saturation was 96% on room air. On neurological exam, he was alert and oriented to person, place and time with intact strength and sensation. No audible murmurs were auscultated on cardiovascular exam. The initial labs were notable for a white blood count of 2.5 × 10^9^/L, hemoglobin of 8.8 gm/dL, and platelet count of 60 × 10^9^/L (baseline 60 × 10^9^/L – 80 × 10^9^/L). Computer tomography (CT) head on day 0 was negative for any acute changes. On day 1, CT chest was performed which revealed interval decrease in size of pulmonary nodules consistent with clinical improvement of pulmonary aspergillosis.

**Fig. 1:**
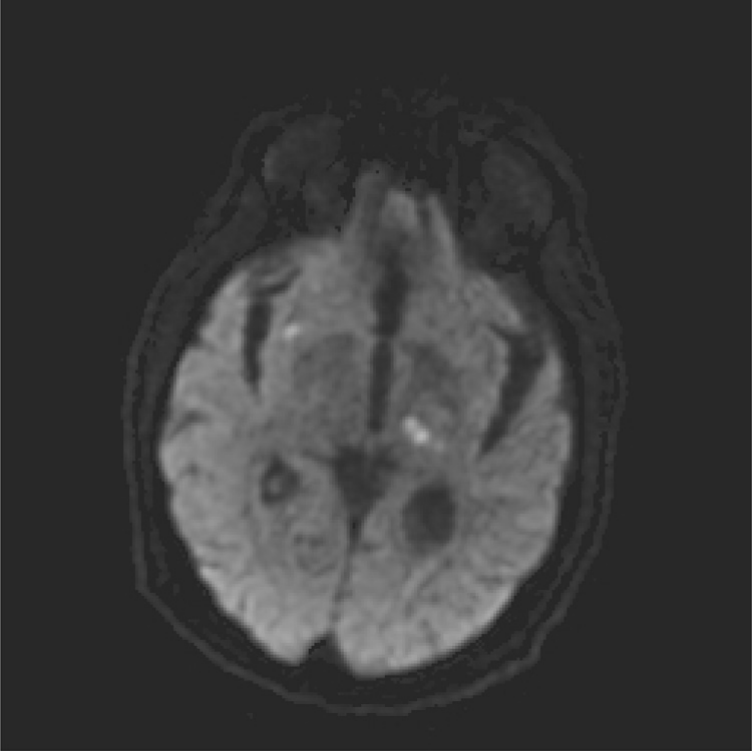
MRI demonstrating bilateral infarcts.

On day 4, the patient became febrile (Tmax >38.2 °C), confused, hypotensive (dropped to 78/50 mmHg from baseline of 90/60 mmHg), with acute right-sided weakness. His cardiac exam was notable for a new harsh holosystolic murmur loudest at the apex radiating to the axilla. Neurological examination revealed lethargy, disorientation, and right lower extremity weakness. A repeat CT head on day 4 was concerning for an acute infarct in the left posterior limb of the internal capsule. On day 5, infectious work-up was initiated and the patient was started empirically on PO posaconazole 300 mg q24 h. Magnetic resonance imaging (MRI) (day 6) revealed multiple acute infarcts including the left thalamus, dorsal midbrain, left superior parietal lobule and right insula concerning for embolic etiology (Fig. 1). Magnetic resonance angiography (MRA) on day 6 also demonstrated stenosis of the left posterior cerebral artery (P2 segment) without aneurysm formation or visible branch occlusion. Lumbar puncture was performed on day 6 and returned negative for infectious etiologies.

**Fig. 2:**
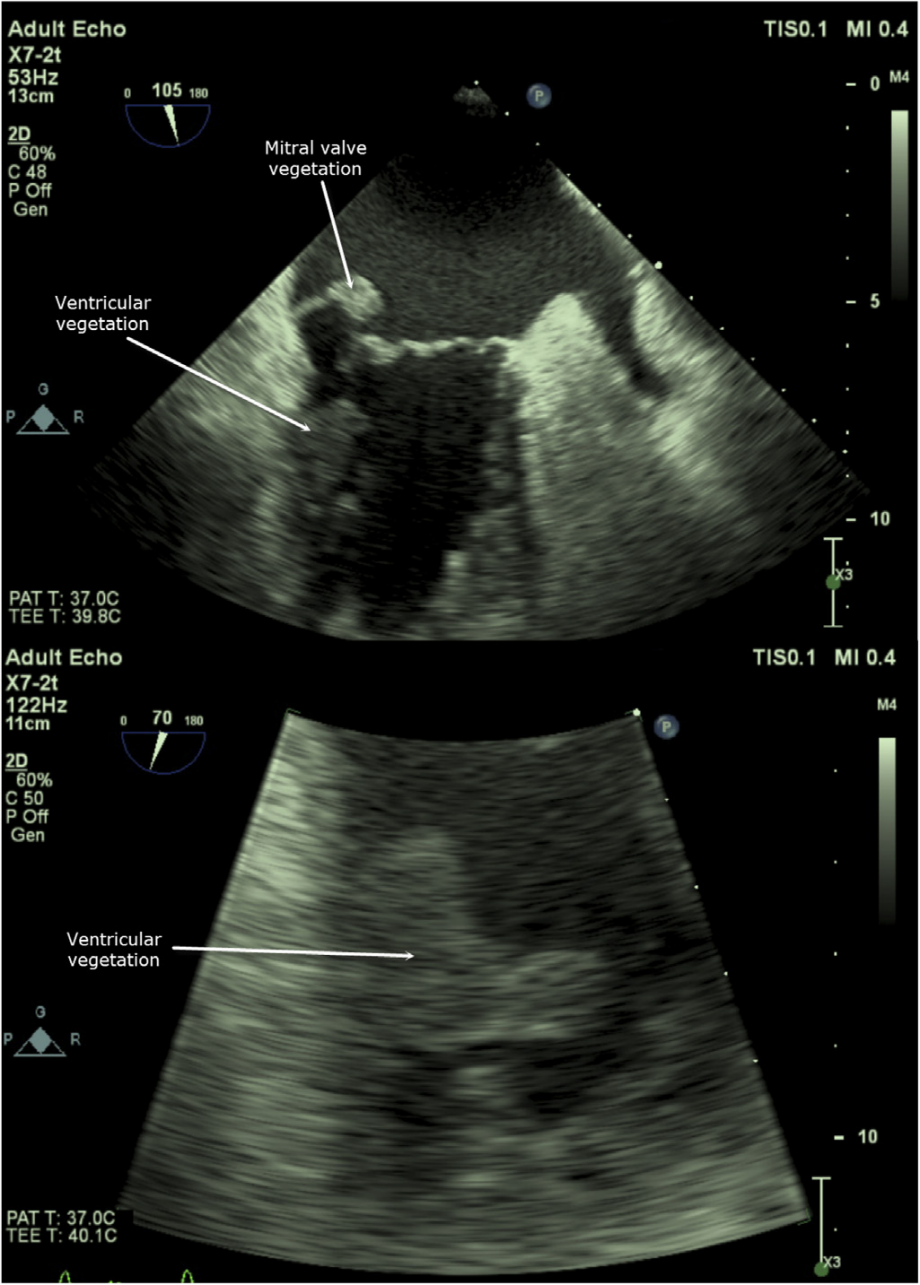
Transesophageal echocardiogram showing a pedunculated mitral valve vegetation and multi-lobed mid-ventricular wall vegetation.

On day 6, IV caspofungin 50 mg IV q24h was empirically added to the regiment of PO posaconzole for concern of culture-negative endocarditis or candidemia. A transthoracic echocardiogram on day 6 revealed a partially visualized mass on a thickened mitral valve. On day 7, serum aspergillus antigen returned positive with an index of 3.69. IV caspofungin and PO posaconazole were discontinued and the patient was started on IV liposomal amphotericin 5 mg/kg q24 h and IV anidulafungin 100 mg q24 h on day 7 based on suspicion of aspergillus endocarditis. Voriconazole and caspofungin were not used based on the patient's acute liver toxicity (ALT 147 U/L, AST 219 U/L). Transesophageal echocardiogram (day 8) confirmed the presence of a pedunculated mobile mass on the atrial aspect of the mitral valve (0.9 × 1.1 cm) as well as a mobile bilobed mass (2 × 2 cm) attached to the anterior mid left ventricular wall and invasion of the myocardium (Fig. 2).

On day 9, the patient developed worsening headache. Repeat CT head on day 9 showed development of subarachnoid hemorrhage (SAH) in the frontoparietal region and evolving subacute infarcts in the posterior limb of the internal capsule and left thalamus. The patient's current antifungal regiment of liposomal amphotericin and anidulafungin was supplemented with IV Posaconazole 300 mg q12 h as salvage therapy based on lack of clinical improvement.

On day 10, Beta-D-glucan assay returned positive (level >500 pg/mL). Serial blood cultures remained negative for bacterial and fungal growth throughout the admission.

Repeat MRI/MRA (day 12) revealed a hypoplastic right vertebral artery and multiple ring enhancing lesions consistent with metastatic lesions. A cerebral angiogram (day 14) revealed two visualized mycotic aneurysms: one in the distal M4 segment frontal branch of right middle cerebral artery (MCA) with localized subarachnoid hemorrhage, and the other in the posterior parietal branch of the left MCA. Several areas of vessel narrowing were also identified on the parietal branch of the MCA and the left posterior cerebral artery consistent with mycotic cerebral vasculitis. The patient underwent an IR-guided obliteration of the right-sided mycotic aneurysm suspected as the source of the subarachnoid hemorrhage. He was not deemed eligible for surgery and per his request, was transitioned to comfort care measures. The patient died on day 16.

## 3 Discussion

Despite advances in medical care, the incidence of infective endocarditis (IE) remains high globally with studies reporting between 1.5 and 9.6 cases per 100,000 with the mortality rate for these cases approximately at 25% [4]. The risk factors for IE have shifted from rheumatic heart disease (less than 5% in the past 2 decades) [4] to nosocomial infections (>25%), degenerative valvulopathies (30–40%), prosthetic valves (20%), and implanted cardiac devices (7%) in high-income countries [5]. Fungal infections comprise the rarest causes of infective endocarditis, accounting for only 1–2% of all cases of IE, with up to 4% of those involving a prosthetic valve [1]. Fungal endocarditis also portends the worst prognosis with mortality rates up to 50% despite aggressive treatment [6].

A literature review of 270 cases from 1965 to 1995 [1] showed patients had prior valve surgery in 74% of all listed cases of fungal endocarditis [7]. Risk for right-sided endocarditis was increased in patients abusing intravenous drugs, receiving total parenteral nutrition, and possessing a central line [8], [9] Other major risk factors include solid organ transplant, bone marrow transplant, and immunosuppression (HIV, neutropenia, corticosteroid therapy, immunosuppressive therapy) [8], [9]. In the described case, the history of HLH, etoposide and steroid therapy, and a history of pulmonary aspergillosis predisposed our patient to develop aspergillus endocarditis.

It is possible the initial therapy with isavuconazole failed to suppress hematogenous dissemination, although there was decrease in size of the patient's previous right-lower lobe nodule. A unique aspect of this case is the development of left-sided endocarditis, despite having risk factors for right-sided endocarditis (PICC line and central line placement). This suggests that pathological factors apart from endothelial trauma, such as vascular tropism of Aspergillus, may play a more significant role in disseminated aspergillosis [8].

The clinical presentation of fungal endocarditis is difficult to distinguish from bacterial endocarditis [2]. Fever (72%), embolic events (69%), new or changing heart murmurs (41%) and sudden vision loss (13%) are common presenting symptoms [3], [9]. The most common organisms are Candida (53–68%) [2] and Aspergillus (20–25%) [2], [3]. Embolization is more common in Aspergillus endocarditis with frequent involvement of brain, kidney, spleen and lungs [2], [9]. In the described case, it was difficult to distinguish between bacterial and fungal endocarditis based on the initial clinical presentation of fever, embolic stroke and a new onset cardiac murmur alone.

There are few reported cases of fungal myocardial invasion in patients with hematological malignancies [10], [11], allogeneic hematopoietic stem cell and bone marrow transplants [3], [12], or with immune dysfunction [13]. Myocardial invasion with invasive aspergillosis in immunocompetent patients has also been described [14]. Only 33 patients with intracranial fungal aneurysms have been reported in the literature between 1995 and 2005 with two originating from the heart, and the majority involving direct invasion (trauma, surgery) or hematogenous seeding (lung, GI tract) [15]. We report an extremely rare case of aspergillus endocarditis with left ventricular myocardial invasion, cerebral vasculitis and intracranial fungal aneurysm formation in a patient with HLH. In our review of the literature, we have not found another reported case of combined ventricular myocardial invasion, intracranial fungal aneurysm formation, and cerebral vasculitis in the setting of Aspergillus infection.

Establishing the diagnosis in fungal endocarditis can be challenging. Blood cultures remain the most important indication of endocarditis [2]. However, the sensitivity of detection of Aspergillus in blood cultures is approximately 4% [2]. In the described case, blood cultures remained negative throughout the admission. Transthoracic echocardiography (TTE) and transesophageal echocardiography (TEE) have been shown to be useful with a combined sensitivity rates of 77% for fungal endocarditis [9] and up to 89% for the detection of Aspergillus endocarditis [9]. Mannan antigen (a component found in Candida spp. cell walls), and anti-mannan antibodies have a combined sensitivity 83% and a specificity of 86% in the detection of Candida endocarditis [2]0.1,3 Beta-D-Glucan, a polysaccharide present in the cell wall of almost all fungi, can be helpful in the diagnosis of fungal endocarditis if above serum threshold levels (>60 pg/mL) [2]. Galactomannan, a component of the cell wall of Aspergillus, can be measured as an index, which if greater than or equal to 0.5 is especially helpful in the diagnosis of Aspergillus endocarditis in high-risk patients (prolonged neutropenia, transplantation, etc.) with a sensitivity and specificity of 100% and 97.5%, respectively [2]. The galactomannan index in our patient was 3.69, and the Beta-D-glucan levels for our patient was over 500, making the probability of infection with Aspergillus high.

Given the high mortality rates seen in aspergillus endocarditis, aggressive management with a combined surgical and medical approach is necessary [9]. Guidelines recommend surgical replacement of an infected valve within 1 week (Class I indication) of diagnosis [2] with concomitant use of an antifungal agent [2] followed by chronic suppressive therapy [9]. For patients who are poor surgical candidates, such as the patient described in this case, induction therapy of antifungals followed by suppressive therapy can be used [2]. Voriconazole is both the first line for induction and long-term suppression therapy for treating Aspergillus endocarditis [2]. However, the hepatotoxic profile of voriconazole prevented its use in our patient with acute liver injury. The second and third line antifungal agents are liposomal amphotericin B (AmB) and posaconazole, respectively [16]. Recently it has been suggested that combination salvage therapy such as voriconazole and anidulafungin may improve the outcome for patients with IA [3]. Since voriconazole was not an option, combination therapy with AmB and anidulafungin was initiated as studies have shown potentially improved patient outcomes with different combinations of salvage antifungal regiments [17]. Posaconazole was added to the antifungal regiment when the patient showed no signs of clinical improvement. Long-term suppressive therapy with azoles is reasonable as fungal endocarditis relapse rates are high [2].

Outcomes associated with Aspergillus endocarditis are poor. In a study by Kalokhe et al., out of 53 case reports of Aspergillus endocarditis from 1950 to 2009, survival rate after a combined surgical and medical approach was 32% and only 4% with antifungal therapy alone [9]. Early detection of fungal endocarditis and empiric antifungal therapy is crucial, especially in immunosuppressed patients who are not improving with empiric antibiotic therapy [9].
